# Influence of Bariatric Surgery on Erectile Dysfunction—a Systematic Review and Meta-Analysis

**DOI:** 10.1007/s11695-023-06572-9

**Published:** 2023-04-22

**Authors:** Piotr Małczak, Michał Wysocki, Magdalena Pisarska-Adamczyk, Jakub Strojek, Hanna Rodak, Ilie Lastovetskyi, Michał Pędziwiatr, Piotr Major

**Affiliations:** 1 2nd Department of General Surgery, Jagiellonian University Medical College, Cracow, Poland; 2 Department of General Surgery and Surgical Oncology, Ludwik Rydygier Memorial Hospital in Cracow, Cracow, Poland; 3 Department of Medical Education, Jagiellonian University Medical College, Cracow, Poland

**Keywords:** Bariatric surgery, Erectile dysfunction, Meta-analysis

## Abstract

**Introduction:**

Obesity is associated with a higher prevalence of various comorbidities including erectile dysfunction (ED). Bariatric surgery leads to weight loss and remission of weight-related diseases. The exact influence of bariatric treatment on ED is yet to be established; however, the number of papers on the subject is growing.

**Methodology:**

A systematic review with meta-analysis comparing erectile dysfunction before and after surgery was conducted according to PRISMA guidelines with a literature search performed in June 2022. Inclusion criteria involved (1) ED assessment using the International International Index of Erectile Function (IIEF) and (2) longitudinal study design. Secondary endpoints involved hormonal changes and specific fields of IIEF.

**Results:**

An initial search yielded 878 records. Fourteen studies were included in the meta-analysis involving 508 patients. The quality of analyzed studies was moderate. Analysis showed significant differences in IIEF before and after surgery (Std. MD = 1.19, 95% CI 0.72 to 1.66, *p*<0.0001). Testosterone after surgery is higher by 156.32 pg/ml (95% CI 84.78 to 227.86, *p*<0.0001). There were differences in erectile function (MD:4.86, *p* < 0.0001), desire (MD: 1.21, *p* < 0.0001), intercourse satisfaction (MD: 2.16, *p* < 0.0001), and overall satisfaction (MD: 1.21, *p* = 0.003). There were no differences in terms of orgasms (MD: 0.65, *p* = 0.06).

**Conclusion:**

There are differences in ED before and after bariatric surgery. Patients achieve 19% more in the IIEF questionnaire showing improvement. Further studies, including multivariate regression models on large cohorts, are required to determine whether the surgery is an independent factor in alleviating ED.

**Graphical Abstract:**

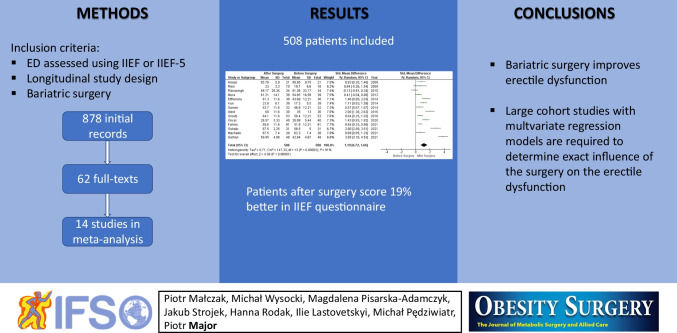

**Supplementary Information:**

The online version contains supplementary material available at 10.1007/s11695-023-06572-9.

## Introduction

Obesity is a well-known condition associated with comorbidities such as diabetes, hypertension, or metabolic syndrome [1]. It also drastically impairs the quality of life which apart from general health is considered an important aspect of a patient’s well-being [2]. There are many elements of health-related quality of life, one of which is sex life [3]. Males with obesity have a greater prevalence of erectile dysfunctions (ED) than patients with a body mass index (BMI) below 30 [4]. This can be caused by many reasons, both psychological and physical [5]. Patients with higher BMI may have lower self-esteem and may not be comfortable with their bodies, thus avoiding sexual contact. From a biological standpoint, an increased percentage of fatty tissue leads to a general state of inflammation, which can cause vascular insufficiency impairing blood flow into the penile. A higher percentage of fatty tissue increases aromatase activity, which converts testosterone causing its decrease. Combined, these issues may lead to erectile dysfunction [5]. Bariatric surgery is an established treatment method for long-term weight loss which also alleviates symptoms of diabetes, hypertension, and other weight-related comorbidities [6]. In recent years, the number of studies analyzing the potential effect of bariatric surgery on ED has been increasing. This study aimed to systematically review the available literature to assess erectile dysfunction before and after weight-loss surgery.

## Methods

### Search Strategy

A search was conducted by three researchers (JS, HR, and IL) in June 2022. Medline, Embase, Cinahl, and Scopus were reviewed with language restricted to English and using the search terms “bariatrics,” “sleeve gastrectomy,” “gastric bypass,” “sleeve resection” and combinations of these with “erectile dysfunction” and “impotence” using the Boolean operators “AND” and “OR.” Reference lists of relevant publications were assessed for additional references. Furthermore, bibliographies from other systematic reviews or meta-analyses on the subject were searched. The search strategy for the OVID platform is presented in Supplementary File 1. Sleeve gastrectomy and gastric bypass were specifically included in the search strategy as they are the most commonly performed bariatric procedures. The systemic review is registered in the PROSPERO database under CRD42022340799.

### Eligibility Criteria

A paper was included when (1) the study concerned adult patients who underwent bariatric surgery, (2) the study assessed erectile dysfunction using the International Index of Erectile Function (IIEF) Questionnaire, and (3) the study compared pre- and post-surgery results in a longitudinal design [7]. The exclusion criteria were (1) ED assessment using a different tool, (2) study design other than longitudinal, and (3) case-matched studies, reviews, and guidelines.

### Outcome Measures

The primary outcome of this systematic review was erectile dysfunction assessed with a validated questionnaire. The most commonly used were IIEF (75-point scale) and its short iteration IIEF-5 (25-point scale) [8]. Secondary outcomes were hormone levels (testosterone, luteinizing hormone, and estradiol) and specific fields of the IIEF: erectile function, orgasm, desire, intercourse satisfaction, and overall satisfaction.

### Study Selection and Data Extraction

**Fig. 1:**
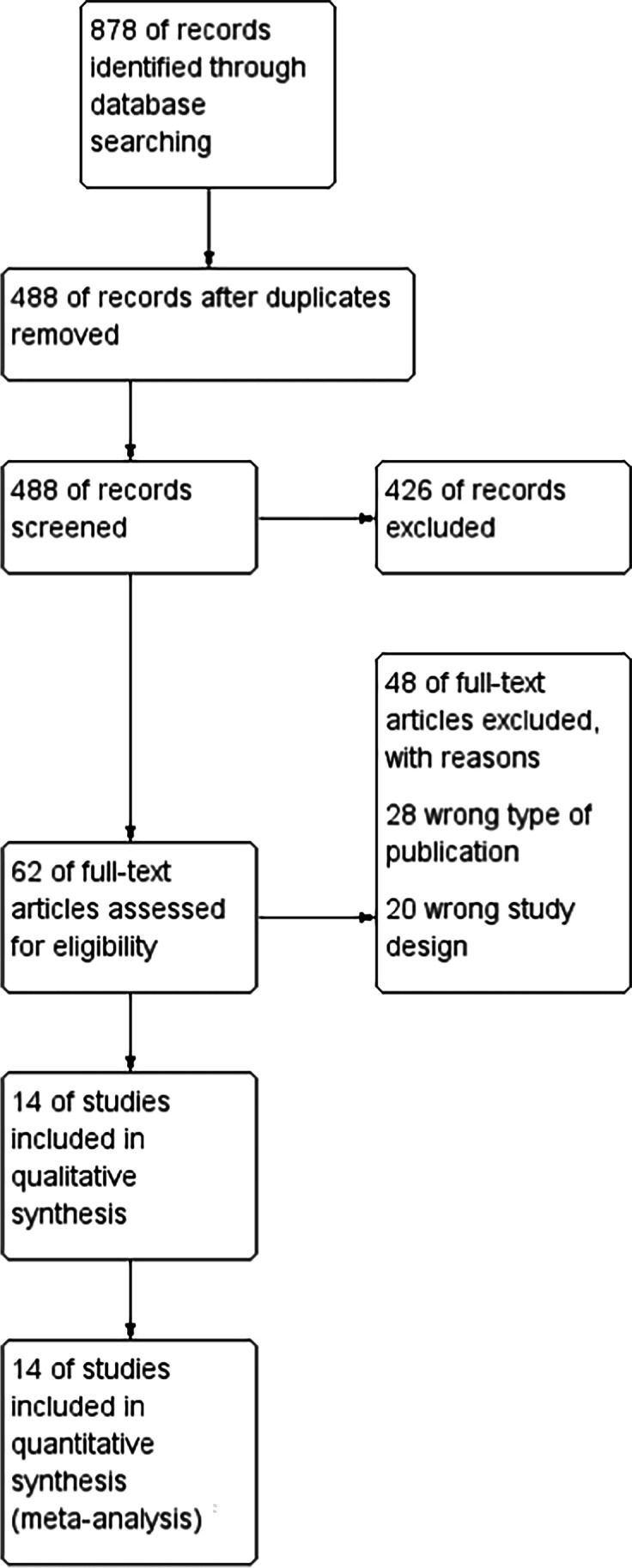
Study selection flow chart

Each of the records downloaded from searches was screened by at least two researchers independently (JS, HR, and IL). The selection was first based on titles and abstracts and then full texts. In case of disagreement, an attempt was made for reaching a consensus within the group. If no resolution was possible, an arbitrary decision was made by the third reviewer. The process of identifying relevant studies is summarized in the PRISMA flow diagram (Fig. 1). Data from included studies were extracted independently by two researchers to a prepared Excel sheet. When available, the following data were extracted: first author, year of publication, number of operated patients, type of surgery, assessment tool, and outcomes of interest (endpoint data).

### Study Quality

The quality of included studies was assessed using the Newcastle-Ottawa Scale, which consists of three factors: patient selections, comparability of the study groups, and assessment of outcomes [9]. This was done in pairs by two review authors (HR, IL). Any disagreements were resolved by discussion or by involving another review author (PM).

### Statistical Analysis

The analysis was performed using RevMan 5.4 (freeware from the Cochrane Collaboration). Statistical heterogeneity and inconsistency were measured using Cochran’s *Q* tests and *I*^2^, respectively. When a study included medians and interquartile ranges, we calculated the mean ± SD using a method proposed by Hozo et al. [10]. Otherwise, when the standard deviation was missing, it was derived as shown by Fu et al. [11]. Standardized mean difference (SMD) and mean difference (MD) with 95% CI are presented for quantitative variables using the inverse variance random-effects method. Statistical significance was observed with a two-tailed 0.05 level for the hypothesis and with 0.10 for heterogeneity testing, while unadjusted *p*-values were reported accordingly. This study was performed according to the Preferred Reporting Items for Systematic reviews (PRISMA) guidelines and MOOSE consensus statement [12, 13].

## Results

The initial search yielded 878 records. After removing duplicates, 488 titles and abstracts were reviewed. Finally, 14 studies were selected and included in the meta-analysis involving 508 patients [14–27]. Patients underwent Roux en-Y gastric bypass, sleeve gastrectomy, gastric banding, banded gastric bypass, one-anastomosis gastric bypass, or biliopancreatic diversion. The follow-up period varied from 6 to 48 months. Three studies reported outcomes in IIEF-5, whereas the rest used IIEF. General characteristics of included studies and patients are presented in Table 1. The quality of included studies was moderate.

**Table 1:** General characteristics of included studies

Author	Year	Study type	Surgery type	Population	IIEF/IIEF-5	Follow-up (months)	Quality NOS
Reis [14]	2009	CS	RYGB	10	IIEF-5	24	7
Araujo [15]	2009	CS	Gastroplasty	21	IIEF	6	7
Ranasingh [16]	2010	CC	GB	34	IIEF	31.79	9
Mora [17]	2012	CS	SG, RYGB	39	IIEF	12	7
Sarwer [18]	2014	CS	RYGB	32	IIEF	48	7
Kun [19]	2014	CS	RYGB	39	IIEF-5	12	7
Efthymiou [20]	2014	CS	SG, RYGB, BPD-DS	30	IIEF	12	7
Groutz [21]	2016	CS	SG	53	IIEF	3	6
Aleid [22]	2016	CS	SG, RYGB, GB	30	IIEF	6	7
Oncel [23]	2020	CS	SG	40	IIEF-5	6	6
Fahmy [24]	2021	CS	SG	81	IIEF	12	6
Machado[25]	2021	CS	SG, RYGB	20	IIEF	18	6
Gokalp [26]	2021	CS	SG	31	IIEF	12	6
Sarhan [27]	2021	CS	SG, SAGB	48	IIEF	12	7

*CC* case-control study, *CS* cohort study, *RYGB* Roux-en-Y gastric bypass, *IIEF* International Index of Erectile Function, *SG* sleeve gastrectomy, *GB* gastric banding, *SAGB* single anastomosis gastric bypass, *NOS* Newcastle-Ottawa Scale

**Fig. 2:**
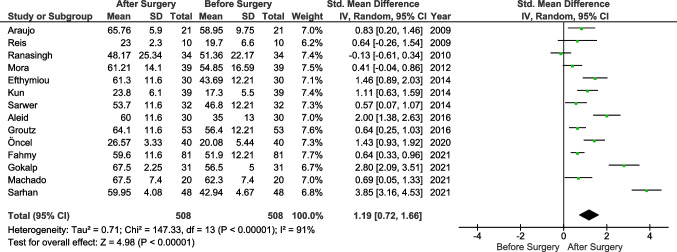
Pooled estimates of the erectile dysfunction comparing before and after surgery. CI confidence interval, df degrees of freedom

**Fig. 3:**
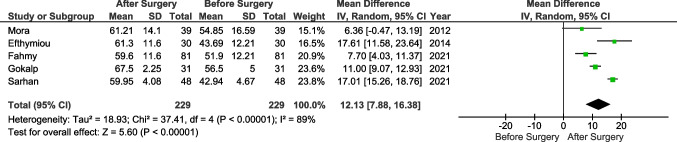
Pooled estimates of the IIEF with 12-month follow-up comparing before and after surgery. CI confidence interval, df degrees of freedom

The primary endpoint (IIEF score) was reported in all studies. Three authors reported results showing no significant differences before and after the surgery [14, 17, 28]. The analysis (Fig. 2) showed significant differences before and after surgery (Std. MD = 1.19, 95% CI 0.72 to 1.66, *p* < 0.0001). The heterogeneity between the studies was very high (*I*^2^ = 91%). Sensitivity analysis showed that 6 studies were the cause of all heterogeneity, and when they were excluded, the result remained significant at *p*<0.0001. Additionally, we performed a subgroup analysis involving studies with a follow-up of 12 months (Fig. 3). Five studies were included with a total of 229 patients. The mean difference was 12.13 in favor of the post-surgery period (MD 12.13, 95% CI 7.88 to 16.38, *p* < 0.0001, *I*^2^ = 89%).

**Fig. 4:**
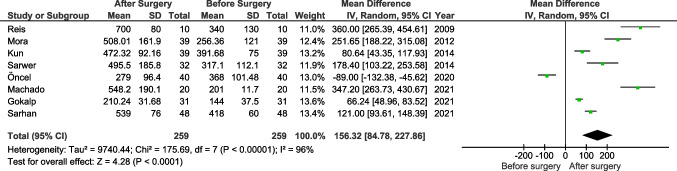
Pooled estimates of testosterone levels comparing before and after surgery. CI confidence interval, df degrees of freedom

Testosterone levels were reported in 8 studies [14, 17, 19, 25–27, 29, 30]. Data were reported in various units and were unified in pg/ml. Sarwer et al. reported that surgery led to lower levels of testosterone; however, the remaining authors showed that the hormone concentration increased [29]. Overall, the analysis showed an increase in testosterone level following bariatric surgery (MD: 156.32, 95% CI 84.78 to 227.86, *p*<0.0001, *I*^2^ = 96%) (Fig. 4).

LH was reported in 4 studies [14, 17, 25, 29]. None of the studies showed significant differences; however, after pooling the data, the analysis showed a significant increase in LH levels (mIU/ml) (MD: 0.66, 95% CI 0.04 to 1.27, *p*=0.04, *I*^2^ = 8%) (Supplementary file 2).

Estradiol was reported in 4 studies [14, 17, 25, 26]. Reis, Gokalp, and Machado reported a significant decrease in estradiol after surgery, whereas Mora et al. did not find any significant differences [17]. Analysis after pooling data showed no significant differences in estradiol levels before and after surgery MD (−10.64, 95% CI −22.44 to 1.16, *p*=0.08, *I*^2^ = 87%) (Supplementary file 3).

Erectile function was reported in 9 studies [17, 18, 21, 24–27, 31, 32]. Sarwer et al. and Machado et al. reported no significant differences whereas the remaining authors showed improvement after surgery [18, 25]. Analysis showed improvement in erectile function after surgery (MD: 4.86, 95% CI 4.10 to 5.63, *p* < 0.0001). The heterogeneity between the studies was low (*I*^2^ = 16%) (Supplementary file 4).

Orgasm was reported in 9 studies [15, 17, 21, 24–27, 29, 32]. The majority of authors showed no differences between the analyzed groups; however, Sarhan et al. and Gokalp et al. presented data showing improvement after the surgery [26, 27]. Meta-analysis did not reveal any statistically significant differences before and after the surgery (MD: 0.65, 95% CI −0.02 to 1.32, *p* = 0.06, *I*^2^ = 88%) (Supplementary file 5).

Desire was reported in 9 studies [15, 17, 21, 24–27, 29, 32]. Analysis showed significant differences between before and after surgery, favoring the latter (MD: 1.21, 95% CI 0.61 to 1.82, *p*<0.0001, *I*^2^ = 87%) (Supplementary file 6). Similar results of the analysis were found in intercourse satisfaction (MD: 2.16, 95% CI 1.44 to 2.88, *p* < 0.0001) and overall satisfaction (MD: 1.21, 95% CI 0.4 to 2.02, p = 0.003) (Supplementary files 7 and 8).

## Discussion

This study aimed to assess erectile dysfunction before and after bariatric surgery. Patients with morbid obesity have a 1.5–3 times greater risk of developing erectile dysfunction in comparison to patients with normal BMI [33]. In general, our meta-analysis showed an improvement in ED when considering the total IIEF score; however, four studies showed no significant effect of the surgery [14, 17, 28, 31]. It is in line with the previous meta-analysis on the subject by Xu et al. and Liu et al., which reviewed the literature up to 2019 [34, 35]. Interestingly, one of the studies included in our review, by Ranasinghe et al., showed results that the surgery did not improve ED but even made it slightly worse [16]. In contrast to previous systematic reviews, our study showed that there are no significant differences in orgasm. In turn, desire and overall satisfaction have been shown to improve significantly.

It is still controversial whether the increased prevalence of erectile dysfunction is caused by hormone disruptions, psychological issues, or vascular conditions. Testosterone levels are decreased by aromatase activity which is increased by the volume of fatty tissue [36, 37]. Our analysis showed that surgery and weight reduction lead to an increase in testosterone levels. An increase in LH additionally shows that the pituitary axis is affected as well. There are numerous psychological aspects affecting a patient’s sexual life, which in general may lead to erectile dysfunction and impaired sexual satisfaction [38, 39]. Morbid obesity causes a higher risk of oxidative stress, which in turn may damage arteries and veins resulting in impaired penile blood flow [40].

Rather than one possible cause of ED alleviation following surgery, such as the increase in testosterone levels, it is a result of a combination of several factors leading to improved general health and mental health. Patients’ sex life is an important aspect of quality of life following bariatric surgery. In our opinion, more emphasis should be placed on this particular disorder, and it should be considered obesity-related comorbidity similar to diabetes or hypertension.

There are several limitations to this study. All of the included studies are observational longitudinal studies. Preferably analysis would involve randomized controlled trials comparing surgery with lifestyle interventions using a longitudinal design, but the number of such studies is scarce. Several of included studies have moderate quality; however, additional sensitivity analysis did not show any change with the removal of these studies. Another limiting factor is the heterogeneity in the operative procedure. Operations that are more malabsorptive may affect the hormonal system differently, resulting in an additional confounding factor.

## Conclusion

There are differences in erectile dysfunction before and after bariatric surgery. Patients achieve 19% more in the IIEF questionnaire showing improvement. Testosterone levels after surgery are significantly higher. Further studies, including multivariate regression models on large cohorts, are required to determine whether the surgery is an independent factor in alleviating ED.

## Supplementary Information

ESM 1 (DOCX 201 KB)

ESM 2 (DOCX 406 KB)

ESM 3 (DOCX 425 KB)

ESM 4 (DOCX 619 KB)

ESM 5 (DOCX 601 KB)

ESM 6 (DOCX 605 KB)

ESM 7 (DOCX 592 KB)

ESM 8 (DOCX 597 KB)
