# Supplementary material for: Influence of Bariatric Surgery on Erectile Dysfunction—a Systematic Review and Meta-Analysis
Source: Obes Surg. 2023 Apr 22;33(6):1652–8. doi: 10.1007/s11695-023-06572-9 (PMC10234872; doi:10.1007/s11695-023-06572-9)
Supplement: Supplementary file 1 — ESM 1 (DOCX 201 KB) [file 11695_2023_6572_MOESM1_ESM.pdf]

1] (bariatric* or gastric by?pass or ryg bor lrygb or roux?en?y* or LSG or sleeve gastrectomy* or SG).ab.,at,kw.

2] (erect* dysfunction or ED or impotence).ab,at,kw.

3] 1 and 2
